# Focused Ultrasound Improves NK-92MI Cells Infiltration Into Tumors

**DOI:** 10.3389/fphar.2019.00326

**Published:** 2019-04-18

**Authors:** Chaopin Yang, Meng Du, Fei Yan, Zhiyi Chen

**Affiliations:** ^1^Department of Ultrasound Medicine, Laboratory of Ultrasound Molecular Imaging, The Third Affiliated Hospital of Guangzhou Medical University, Guangzhou, China; ^2^Experimental Center, The Liwan Hospital of the Third Affiliated Hospital of Guangzhou Medical University, Guangzhou, China; ^3^Paul C. Lauterbur Research Center for Biomedical Imaging, Institute of Biomedical and Health Engineering, Shenzhen Institutes of Advanced Technology, Chinese Academy of Sciences, Shenzhen, China

**Keywords:** natural killer cells, IL-2, focused ultrasound, microbubbles, ovarian cancer

## Abstract

The efficiency of natural killer (NK) cells, adoptively transferred, for treatment against solid tumors is hindered by their difficulty to enter tumors from the blood circulation as well as their inability to prolong viability in the absence of IL-2. Among different sources of NK cells, we used genetically modified NK-92MI cells, a suitable candidate which can release IL-2 to maintain their viability and overcome undesirable side effects caused by systemic administration of exogenous IL-2. In this study, we evaluated whether the combination of focused ultrasound (FUS) and microbubbles can improve adoptively NK-92MI cell infiltration into ovarian tumors through biodistribution, immunofluorescence, and flow cytometry. The treatment effects of using this strategy twice a week were explored. The potential molecular mechanism of FUS assisting NK cell therapy was also initially explored through evaluating the expression of ICAM1 and CX3CL1 by qRT-PCR. Our results indicated that FUS and microbubbles can improve NK-92MI cells’ infiltration into tumors, and the combination of FUS and NK-92MI cells had a better treatment effect compared to the PBS group, but not compared to the NK-92MI group. The qRT-PCR results also showed that CX3CL1 may be involved in the process of FUS-assisted NK cell infiltration. These results indicate that further optimization of the FUS-assisted strategy is still needed to achieve therapeutic benefit.

## Introduction

Natural killer (NK) cells are the first line of the body’s defense against tumors and play critical roles in tumor cell immunotherapy (Cheng et al., 2013; Davis et al., 2015). Specifically for tumor therapy, there are various sources of NK cells that can be used for NK cell therapy, such as iPSC-derived NK cells, peripheral blood mononuclear cells, cord blood-derived NK cells and NK cell lines (NK-92 and NK-92MI). Among them, NK-92MI is a preferable choice because it is “off-the-shelf,” homogenous and easy to expand to satisfy the clinical demands. NK-92MI cells can secrete sufficient quantities of bioactive IL-2, a pivotal cytokine which has proved to be able to activate NK-lymphocytes and enhance immunity against cancer as well as to proliferate and mediate the antitumor effects in the absence of exogenous IL-2. Normal NK cell adoptive therapy often needs intravenous IL-2 to maintain vitality, but administration of high dose IL-2 intravenously will cause serious side effects including fever, chills, hypotension, or tachycardia (Ardizzoni et al., 1994; Kilbourn et al., 2015; Mao et al., 2015), while low-dose IL-2 efficacy is limited by the short half-life (less than 10 min) *in vivo* (Donohue and Rosenberg, 1983). Therefore, stable expression of the IL-2 transgene in NK cells can improve their therapeutic potential in tumor-bearing hosts and avoid the above side effects.

Natural killer cell immunotherapies have proved to be effective for some hematologic malignancies such as acute myeloid leukemia, but the anti-tumor effect of NK cell therapy against solid tumors remains poor. One main reason is the inadequate homing of infused NK cells to the tumor site. It has been demonstrated that increasing the number of NK cells in tumors yields a better prognosis for certain cancers (Ishigami et al., 2000; Murray and Lundqvist, 2016). Patients with a high level of NK cell infiltration were often also found to have a better prognosis than those with a low level of NK cell infiltration (Ishigami et al., 2000; Mamessier et al., 2011; Rusakiewicz et al., 2013; Gras et al., 2015). Therefore, it is important to boost the homing potential of adoptively transferred NK cells.

Ultrasound-mediated microbubble destruction (UTMD) is a promising method that could enhance the release of drugs, genes, nanoparticles, and even cells from vasculature to tumor tissues. The interaction of ultrasound with the microbubbles in vessels can affect the integrity of the tight junctional-complexes through opened intercellular clefts or can stimulate the tumor vasculature for transcytosis, being particularly useful for drug and gene delivery into target tissue (Sheikov et al., 2004, 2008; Deng et al., 2012; Lammertink et al., 2015). The UTMD technique was the most applied technique in drug delivery; for example, Yan et al. (2013) used PTX-liposome loaded microbubbles to increase PTX fourfold in 4T1 tumors. Burke et al. (2014) used 5FU-NP-loaded microbubbles to have twofold decrease in tumor volumes and improve survival compared to 5FU alone. UTMD was also widely used to open the blood-brain barrier and promote drug and gene delivery to the brain (Burgess and Hynynen, 2016). Lin used UTMD to open the blood-brain tumor barrier and successfully deliver liposome consisted of luciferase and glial cell line-derived neurotrophic factor (GDNF) genes to the brain (Lin et al., 2016). Fan et al. (2016) also used cationic microbubbles to load GDNF genes combined with focused ultrasound (FUS) to gain a neuroprotection effect in a Parkinson’s disease xenograft. Arvanitis et al. explored the delivery of two anticancer drugs (doxorubicin and ado-trastuzumab emtansine) into an orthotopic xenograft model of breast cancer brain metastasis using UTMD. Sevenfold and twofold increases for these two drugs in tumor growth were observed compared to the non-FUS group (Arvanitis et al., 2018).

Besides drug and gene delivery, a few reports have also shown the potential of UTMD in favoring immune cell delivery. Alkins et al. (2013a,b) demonstrated that using MRI-guided FUS and microbubbles can deliver targeted NK-92 cells to the desired regions of the brain. Early intensive treatment (daily treatments in the first 5 days) with targeted NK-92 cells and ultrasound could improve long-term survival in 50% of subjects compared with either treatment alone. Sta et al. (2015) also used low dose FUS with microbubbles (ldbFUS with 0.50 MPa peak acoustic pressure) to facilitate the targeting and accumulation of NK cells in a mouse xenograft of human colorectal adenocarcinoma NSG mice in the presence of an anti-CEA immunocytokine (ICK), hT84.66/M5A-IL-2 (M5A-IL-2).

To improve the anti-tumor effect of NK cells for ovarian tumors through enhancing the numbers of NK cells into tumors and to prolong viability of NK cells without administration of exogenous IL-2, in this study, we investigated whether UTMD can assist adoptive NK-92MI cells to accumulate into ovarian tumors from blood vessels, and further explored the treatment effects of the combination of UTMD and NK-92MI cells in ovarian tumor xenograft.

## Materials and Methods

### Mice

The Institutional Animal Care and Use Committee (IACUC) of Guangzhou Medical University approved this research study. All procedures were approved by the IACUC. NOD-Prkdc^em26^II2rg^em26^/Nju (NCG) female mice (4–6 weeks old from Nanjing Biomedical Research Institute of Nanjing University, NBRI) were subcutaneously injected with SKOV3 tumor cells combined with matrigel matrix (Corning, United States) in a ratio of 1:1 (5 × 10^6^ cells in 0.2 ml) at the right flank site. Animals were treated approximately 14 days post-implantation, when the tumors reached 50–100 mm^3^.

Twenty animals with SKOV3 tumors (5 animals for each group) were grouped as follows: (group 1) PBS, (group 2) FUS, (group 3) NK-92MI, and (group 4) FUS and NK-92MI cells. On the day of treatment, animals were assigned to receive PBS, FUS, NK-92MI and a combination of FUS and NK-92MI cells. For (group 2), the mice received intravenous injection of 100 μl Usphere^TM^ Trans+ microbubbles (Trust Bio Sonics, Taiwan). For (group 4), NCG mice had NK-92MI cells injected immediately prior to FUS interacted with microbubbles. The mice were treated twice a week. Tumor volume and the mice weight were measured every time before treatment. The tumor volume was calculated using the formula V = (1/2 × length × width × width) before each treatment.

### Cells Lines

NK-92 and NK-92MI (which was virally transduced to stably express IL-2) cells were purchased from ATCC. NK-92MI cells were maintained in MEMα (Invitrogen, Carlsbad, CA, United States) supplemented with 12.5% horse serum (gibco, United States), 12.5% fetal bovine serum (gibco, United States), 0.2 mM inositol, 0.1 mM β-mercaptoethanol, 0.02 mM folic acid and penicillin/streptomycin. NK-92 was cultured based on the NK-92MI culture medium, and 100–200 U/ml recombinant IL-2 was added. Human ovarian cancer cell lines SKOV3 were maintained in DMEM basic supplemented with 10% fetal bovine serum and penicillin/streptomycin.

### Cellular Cytotoxicity Assay

Natural killer cell-mediated cellular cytotoxicity was determined using a non-radioactive cellular cytotoxicity assay kit (Promega, United States). In a round bottom 96-well plate (Corning Inc.) 100 μl of NK-92MI cells at effector-to-target ratios (means ratios of NK-92MI cells to SKOV3 cells) of 1:1, 2:1, and 5:1 were incubated at 37°C with 5% CO_2_ for 6 h. The target spontaneous release (SKOV3), culture medium background, target maximum release (add lysis solution to SKOV3 before 45 min from the end of the incubation) and volume correction control (10 μl lysis solution and 100 μl culture medium) were operated according to the manufacturer’s instructions. After the incubation, the 96-well plate was centrifuged at 250 *g* for 2 min, the supernatants (100 μl each) were removed to a new flat bottom 96-well plate for detection. The absorbance was read in microplate at 490 nm after the end of the reactions. All experiments were performed in triplicate. Specific lysis (%) was calculated as % Cytotoxicity = (Experimental spontaneous-Effector spontaneous-Target spontaneous)/(Target maximum-Target spontaneous) × 100%.

### IFN-γ and IL-2 Release Assay

The total of 1 × 10^6^ of NK cells per well were co-incubated with SKOV3 cells in 96-well plates at ratios of 1:1, 2:1, and 5:1 for 6 h at 37°C. The culture supernatants were assayed for IFN-γ or IL-2 secretion by enzyme-linked immunosorbent assay (ELISA) using a kit from R&D Systems according to the manufacturer’s protocol. Data depicted in figures represent mean values of triplicate wells from one of three representative experiments with similar results.

### Flow Cytometry to Detect NK Cells Number in Peripheral Blood and Tumor

The following antibodies were used: CD56-PE, CD45-APC, CD3-PercpCy5.5, all from Becton Dickson. Flow cytometry was done on a BD FACS Aria^TM^ III and data were analyzed using FlowJo software (BD).

Briefly, venous whole blood was collected into vacutainer tubes containing heparin. Then 100 μl whole blood was transferred into epoxy epoxide tube and incubated with CD56-PE, CD45-APC, CD3-PercpCy5.5 for 30 min in dark. Then 100 μl whole blood was added to 2 ml BD Lysing Buffer to lyse red blood cells for 15–30 min until solution clarification. The whole blood was further centrifuged in 500 g for 5 min. Then, the cell precipitation was washed. The solution was replaced with 350 μl PBS. Samples were collected on BD FACS Aria III cytometer, and the data were analyzed using FlowJo software.

Tumors were minced with a razor blade as much as possible, then incubated in collagenase IV (Sigma: #C5138), 0.1 mg hyaluronidase (Diamond:A005477), and 200 U DNase I (Sigma:#D5025) DPBS with Mg^2+^/Ca^2+^ at 37°C for 1–2 h to make single cells. Then the mixture was stained with antibodies against human CD56-PE, CD45-APC, and CD3-PercpCy5.5. Samples were collected on BD FACS Aria^TM^ III cytometer, and data were analyzed using BD FACS Aria^TM^ III Cytometer software and FlowJo software.

### RT-qPCR

Total RNA was extracted from tumor tissues using the RNeasy Kit (Servicebio, China). The primers for the analysis were synthesized by Servicebio. The primer sets used arethe following: GAPDH: F, 5′-ACTTTGGTATCGTGGAAGGACTCAT-3′, R, 5′-GTTTTTCTAGACGGCAGGTCAGG-3′; CX3CL1: F, 5′-GGGAATGGACGAGTCTGTGG-3′, R,3′-ACGGGAGGCACTCGGAAAA-5′; ICAM1: F, 5′-CCGTTGCCTAAAAAGGAGTTGC-3′, R, 3′-TGGCAGCGTAGGGTAAGGTTC-5′. Quantitative real-time PCR was performed using fluorescent dye SYBR green Supermix according to the manufacturer’s instructions. Amplification was performed on a Bio-Rad FFX96 Real-time System. GAPDH was used as an internal control and the ΔΔCT method was used to calculate changes in fold expression as fold increase (2^-ΔCT^), where ΔCT = CT (Target gene)-CT (GAPDH).

### Histological Analysis

Animals were sacrificed by excessive anesthesia. Tumors were removed and fixed in 10% paraformaldehyde. Prior to cutting sections, the block was allowed to equilibrate to 20°C. Tissue blocks were sectioned at 18 μm thickness. Sections from the same blocks were also stained for anti-CD45 fluorescence markers (546 nm) and DAPI. Images from sections were captured using Zeiss upright widefield microscope and ZEN software. Mice that received Dir-NK cells underwent the same procedure for tissue preparation and sectioning. Immunohistochemistry results are observed using the confocal microscopy. The Cy3 fluorescence of CD45 expression (mouse anti-human, 1:200, BD, United States) represents the NK-92MI cells.

### Cardiotoxicity

The heart tissues were harvested after the last injection over 1 week and fixed in 4% paraformaldehyde. The tissues were frozen and then cut into sections and mounted onto glass slides, then stained with H&E. Finally, the H&E-stained sections of heart tissue were imaged with light microscopy (Nikon Ti, Japan) and examined for safety problems by FUS and NK-92MI cells. Cardiotoxicity was defined as myofibrillar loss and disarray, as well as cytoplasmic vacuolization.

### FUS Therapy System and *in vivo*FUS Therapy

A focused transducer (1.0 MHz, IBG0112, ndtXducer, United States; SF = 1.75″, Diameter 38 mm) was used for all ultrasound exposures. The system consists of a function generator Tektronix AFG3102C drove 120 mV (cycles 100, interval 1 ms) and an Amplifier Research (AR) RF amplifier 200A400A is the model of amplifier 60%–a transducer is capable of delivering focused and spatiotemporally controlled ultrasound energy. An ultrasound test tank system (Precision Acoustics Ltd., United Kingdom) equipped with a hydrophone (2010, Precision Acoustics Ltd., Dorchester, United Kingdom) in degassed water was used to calibrate the negative peak rarefication pressure of the focused transducer. It was found that the focused transducer could deliver 0.5 MPa in 10% duty cycle if set up according to the parameters above.

**FIGURE 1:**
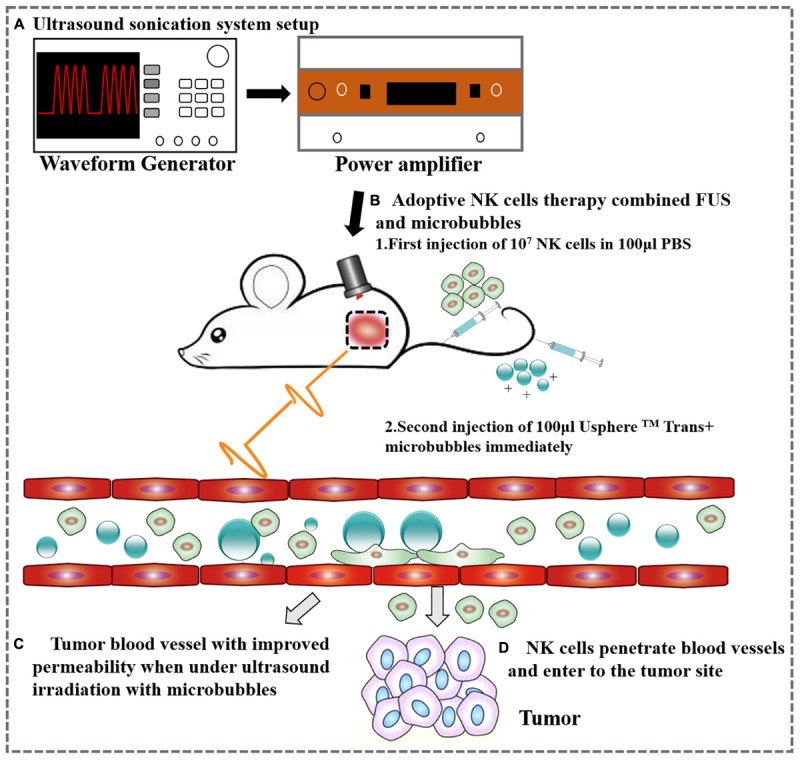
A schematic representation of the ovarian cancer treatment with FUS and NK cells. **(A)** Ultrasound sonication system setup. **(B)** Adoptive therapy of NK cells. **(C)** Tumor blood vessel with improved permeability after ultrasound irradiation. **(D)** NK cells extravasate from the tumor vessel following FUS.

The mouse was placed on the table and the coupling cone was positioned on the tumor with ultrasound transmission gel. The transducer was used to scan the tumor manually at its frequency (1 MHz) for 10 ms every second for 1 min. During the 1 min of ultrasound, 100 μl USphere^TM^ Trans^+^ microbubbles (0.8–1.5 μm, 1–4 × 10^10^ particles/ml) and 100 μl of 10^7^ NK cells were delivered via a tail vein catheter (Figure 1). The USphere^TM^ Trans^+^ microbubbles were bought from Trust Bio Sonics, with a diameter of 0.8–1.5 μm and a concentration of 2–6 × 10^10^ particles/ml–the potential was +40∼+50 mV. The microbubbles were diluted 1.5 times with PBS before used.

### Statistical Analysis

All graphs were created using GraphPad Prism 6 (GraphPad Software Inc.), and the statistical analyses were carried out using SPSS software (version 22.0, SPSS, Chicago, IL, United States). The cellular cytotoxicity assays of NK cells against SKOV3 were repeated three times. The IFN-γ and IL-2 release assays were also repeated at least three times for at least three independent samples. The standard deviations were indicated as error bars in each graph, and the data were analyzed by Student’s *t*-test. The results of tumor volumes and mice weight were analyzed with one-way repeated measures ANOVA to compare different treatment effects in different groups. *P*-values less than 0.05 (with asterisk) were considered statistically significant, and no asterisk meant the result was not significant. The continuous data were presented as mean ± SEM. Data from all experiments were representative of at least three experiments unless otherwise indicated.

## Results

### NK-92MI Cells Have Stronger Cytotoxic Effects Against SKOV3 Cells *in vitro*

**FIGURE 2:**
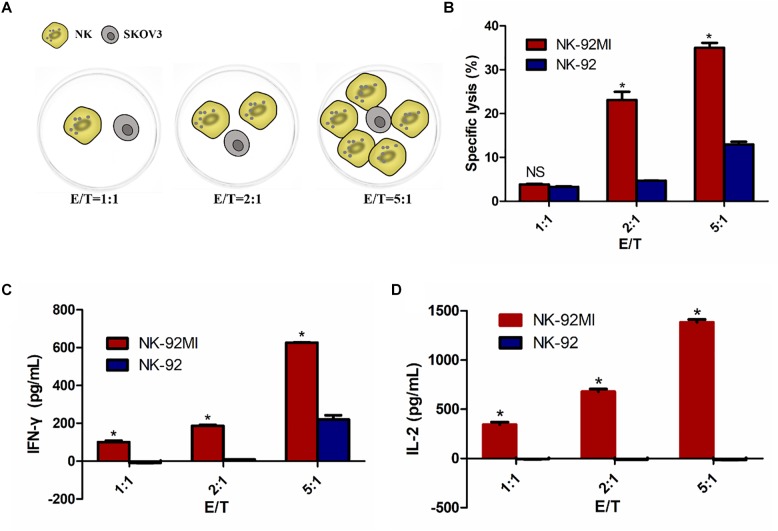
NK-92 and NK-92MI cytotoxicity assay on ovarian cancer cells SKOV3. **(A)** Schematic representation of NK cells against SKOV3 cells. E/T, effector (NK cells) to target (SKOV3 cells) ratio. **(B)** Cell killing by NK-92 and NK-92MI were investigated after co-incubation with SKOV3 cells as targets for 6 h at different effector to target ratios. **(C)** IFN-γ release by NK-92 and NK-92MI in the presence of SKOV3 using a standard ELISA assay. **(D)** IL-2 release by NK-92 and NK-92MI in the presence of SKOV3 using a standard ELISA assay. Statistical analysis by Student’s *t*-test.^∗^*P* < 0.05 was considered statistically significant.

To determine whether IL-2 expression could confer NK-92 with enhanced IFN-γ production and cytolytic activity, we assessed cytotoxicity of NK-92MI and NK-92 cells against SKOV3 cells using an LDH assay at varying ratios of effector cells to target cells (1:1, 2:1, and 5:1). Cell lysis was proportional to the ratios of effector-to-target cells, consistent with both previously published reports (Li et al., 2017; Zhang et al., 2017). The results showed significantly enhanced cytotoxicity of NK-92MI cells against SKOV3 cells compared with NK-92 cells (Figure 2).

There was 34.51% specific lysis at an E/T ratio of 5:1 for NK-92MI cells (Figure 2B). A corresponding increase in the expression of IFN-γ and IL-2 at an E/T ratio of 5:1 for NK-92MI cells was observed–648.40 and 1383.37 pg/ml, respectively (Figure 2C,D). In contrast, there was no IL-2 secretion for NK-92 cells (Figure 2D).

### FUS Can Enhance Accumulation of NK Cells in Ovarian Cancer Xenograft

**FIGURE 3:**
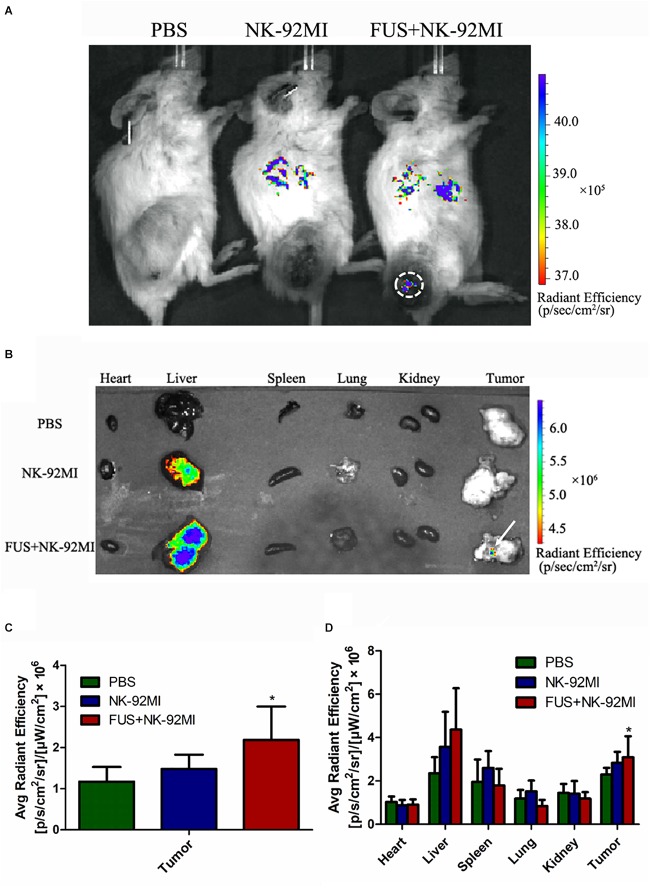
Biodistribution of NK-92MI in xenograft ovarian tumor model. **(A)** NCG female mice were subcutaneously injected with SKOV3 cells. On day –14, the tumors were allowed to grow to 70–100 mm^3^. Mice were randomly divided into three groups (PBS, NK-92MI, and FUS+NK-92MI). Then, animals were treated by intravenous injection of 10^7^ NK-92MI stained with DiR dye. The biodistribution of NK-92MI was assessed with Caliper Spectrum IVIS system. **(B)** Special DiR tissue fluorescence for each organ was quantified using the Caliper Spectrum IVIS system. **(C)** Quantification analysis of fluorescent intensity in the PBS, NK-92MI and FUS+NK-92MI groups from panel **(A)**. The tumor site of the FUS+NK-92MI group has stronger fluorescent intensity compared with the PBS and NK-92MI groups. **(D)** Quantification analysis of fluorescent intensity from different organs in panel **(B)**. The tumor site of the FUS+NK-92MI group has stronger fluorescent intensity compared with the PBS and NK-92MI groups. ^∗^*P* < 0.05 was considered statistically significant.

**FIGURE 4:**
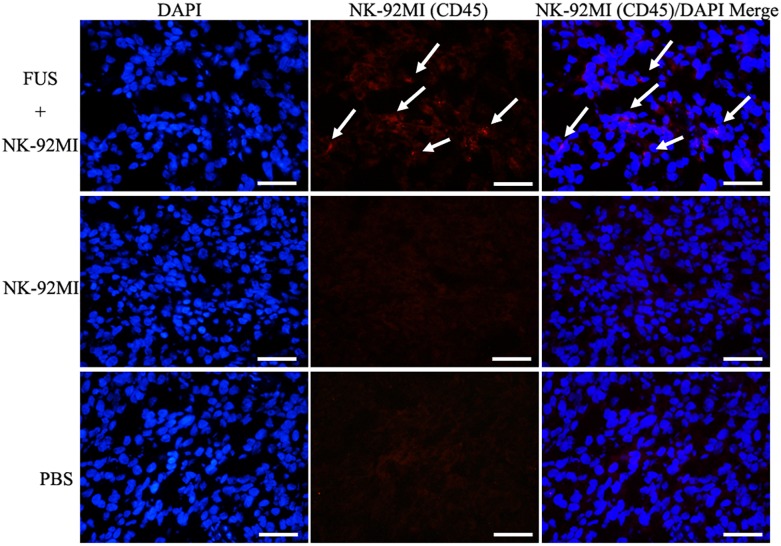
Immunofluorescence evaluation of NK-92MI cells in tumor. Anti-CD45-Cy3 (Red) staining of tumor regions show accumulation of NK-92MI cells (white arrows) in the FUS+NK-92MI group. Nuclei were stained with DAPI (Blue). Scale bar: 50 μm.

We performed a biodistribution study to determine whether FUS can enhance NK cells homing to the tumor site. The fluorescent dye lipophilic carbocyanine DiOC_18_ (Murray and Lundqvist, 2016) (DiR) was used to label NK-92MI cells and to track their presence *in vivo*. Mice were intravenously injected with PBS, and NK-92MI cells were stained with DiR or the combination of FUS, and NK-92MI cells were stained with DiR. The DiR-labeled NK cells *in vivo* were imaged at 24 h after the injection by IVIS Spectrum. The *in vivo* imaging results showed that the FUS + NK-92MI cell group had more NK-92MI cells compared to the NK-92MI and PBS groups in the tumor sites (*P* < 0.05) (Figure 3A,C). The DiR fluorescence for each organ was also quantified using the IVIS Spectrum imaging system, and the majority of NK-92MI cells accumulated in the liver. Nevertheless, the bioluminescence signals of NK cells in the tumor were detectable in the FUS+NK-92MI group and had much stronger signals compared to the NK-92MI and PBS groups (*P* < 0.05) (Figure 3B,D). These results could be further verified by immunofluorescence with anti-CD45-Cy3 (red) and DAPI (blue) (Figure 4).

**FIGURE 5:**
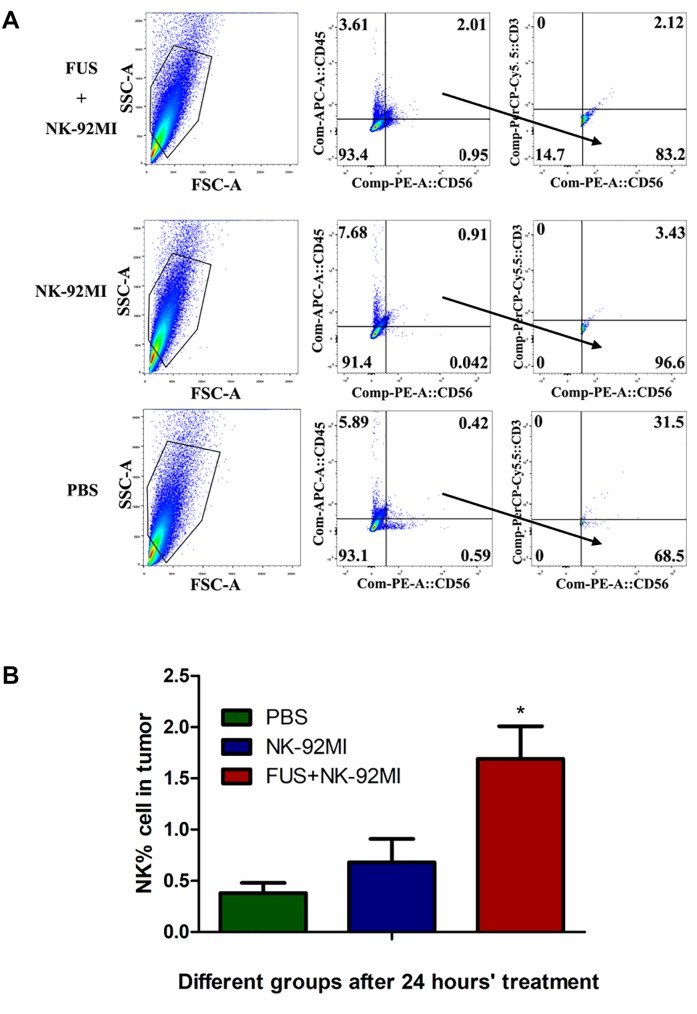
Flow cytometry evaluation of NK-92MI cell numbers from single tumor cell suspension in different treatment groups. **(A)** Single tumor cell suspension of different groups was assessed through flow cytometry. The CD56+CD45+CD3- cells were recognized as NK cells. **(B)** The number of NK-92MI cells in the FUS+NK-92MI group was significantly higher than those in the PBS and NK-92MI groups. ^∗^*P* < 0.05 was considered statistically significant (*n* = 3 for each group).

The NK cells in tumor tissue after 24 h were also quantified with CD45+CD56+CD3- through flow cytometry, and about 1.69 ± 0.32% NK-92MI in all cells were detected in tumor tissue (Figure 5). The number of NK-92MI cells into the tumor was increased 2.5-fold compared to the NK-92MI cell group with the combination of FUS (Figure 5B). No NK cells in the blood were detected.

### Tumor Progression

**FIGURE 6:**
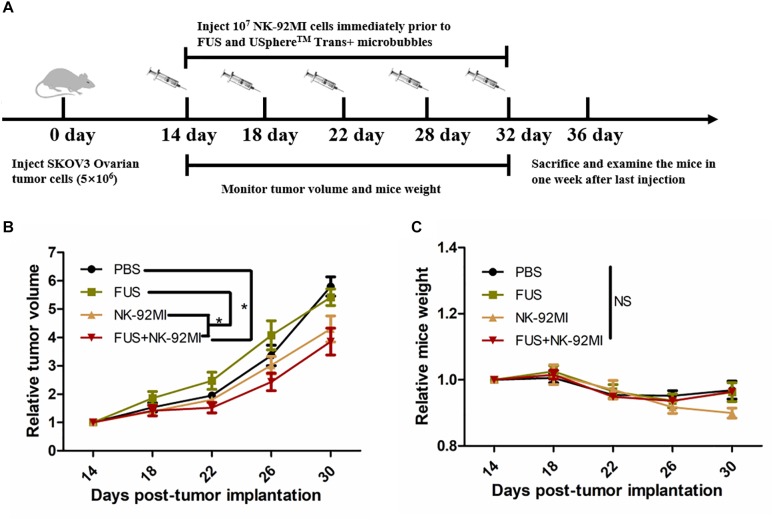
FUS delivery of NK-92MI cells inhibits SKOV3 tumor growth. **(A)** Schematic of *in vivo* studies using FUS interacted with microbubbles assisted adoptive NK cells to treat SKOV3 tumors in a mouse xenograft NCG mice. **(B)** Relative tumor volumes in NK-92MI cell-based treatment. **(C)** Relative mice weight in NK-92MI cells-based treatment, respectively. Statistical analysis by repeated measure ANOVA. ^∗^*P* < 0.05 was considered statistically significant (*n* = 5 for each group).

Totally, only tumor volumes in the FUS+NK-92MI group were statistically smaller than the PBS group (*P* < 0.05) (Figure 6). However, the tumor volumes in the FUS+NK-92MI group were not significantly smaller than the NK-92MI group. More NK-92MI cells did enter into the tumor with the assistance of FUS compared to the NK-92 MI group, which was proved previously by immunofluorescence and flow cytometry results in this study. These conflicting results may be explained by the fact that the NK cells that entered into tumor still had not reached a high enough number to work effectively in the FUS+NK-92MI group. Another possibility could be that the NK cells that entered the tumor were suppressed by the tumor microenvironment. It was not expected that the tumor volumes in the NK-92MI or FUS+NK-92MI groups were statistically smaller than the FUS group. It was intriguing that tumor volumes in the FUS group appeared to have a faster growth rate than the PBS group initially.

There were no significant differences in mice weight in different groups (*P* > 0.05) (Figure 6C). However, the mice weight in the FUS+NK-92MI group appeared to have a weight loss compared with other groups (although without significant differences), which indicated that the FUS+NK-92MI group may undertake the anti-tumor effect.

### The Relationships Between FUS Treatment and ICAM1 and CX3CL1 Expression

**FIGURE 7:**
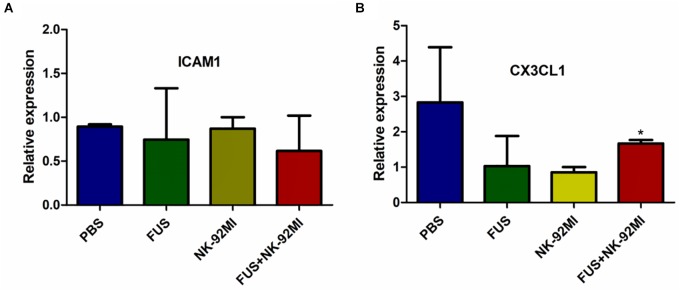
qRT-PCR analysis of ICAM1 and CX3CL1 expression 24 h after treatment. **(A,B)** ICAM1 and CX3CL1 expression 24 h after treatment, respectively. No relative expression of ICAM1 was observed. CX3CL1 expression in the FUS+NK-92MI group was higher than in the NK-92MI group, but not different from PBS and FUS group. ^∗^*P* < 0.05 was considered statistically significant.

In this study, we used RT-qPCR to evaluate the expression of intercellular adhesion molecule 1 (ICAM1) and chemokine (C-X3-C motif) ligand 1 (CX3CL1). We investigated whether the effect of FUS-combined microbubbles could induce ICAM1 expression. ICAM1 plays an important role for tissue homing and residency within ICAM-rich endothelial vessels. Our results showed that ICAM1 expression in the PBS, NK-92MI and FUS+NK-92MI groups 24 h after treatment had no differences between each other (Figure 7A). Interestingly, we found that the CX3CL1 expression of the FUS+NK-92MI group was significantly higher than the NK-92MI group 24 h after treatment (*P* < 0.05) (Figure 7B), which meant that the combination therapy can stimulate CX3CL1 expression.

### Cardiotoxicity Results

**FIGURE 8:**
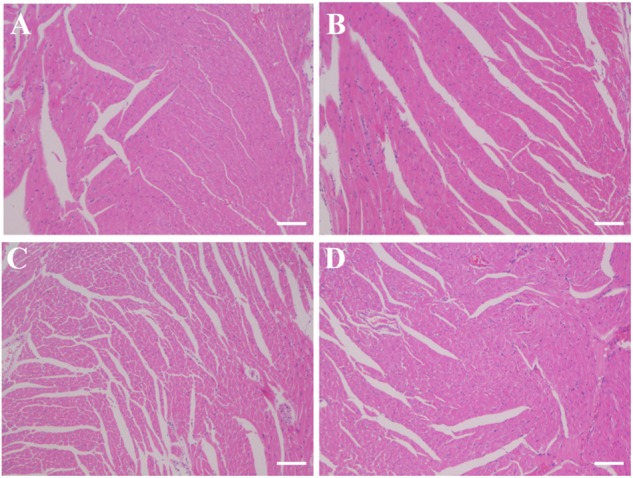
Histology slides for cardiac toxicity in different treatment groups **(A)** PBS group; **(B)** FUS group; **(C)** NK-92MI group; **(D)** FUS+NK-92MI group). Cardiac tissues were fixed and frozen, and sections were mounted on glass slides. The frozen sections were stained with H&E and examined by light microscopy for morphological analysis. Only one representative image from each group was shown. Scale bar: 100 μm.

No damage of the cardiac tissues in any of the treatment groups was observed (Figure 8).

## Discussion

In this study, we demonstrated that FUS interacting with microbubbles could promote intravenous NK-92MI cells to enter a tumor. The results of flow cytometry using CD56^+^CD45^+^CD3^-^ revealed that the ratio of NK-92MI to tumor cells in the tumor was 1.69 ± 0.32%, which was similar to two other studies the NK cell to brain tumor cell ratio in the blood-brain barrier (BBB) opening with UTMD is 1:100 (Alkins et al., 2013a) whereas the NK cell to human colorectal adenocarcinoma cell ratio in blood tumor barriers opening with UTMD is 1.21 ± 0.32% (Sta et al., 2015).

The mechanism of how FUS and microbubbles facilitated cells to accumulate into the tumor is still not clear. Most studies focusing on the BBB opening believed that it was the sterile inflammatory response (SIR) caused by stable oscillations or possibly inertial cavitation, which increases the cytokines, chemokines, trophic factors (CCTF) and cell adhesion molecules (CAM) expression in the parenchymal microenvironment (Gadani et al., 2015; Kovacs et al., 2015). These cytokines can attract immune cells to the inflammatory sites (Maghazachi, 2010; Czaja, 2014; Kondo et al., 2015). In these studies, we used qRT-PCR to assess ICAM1 and CX3CL1 expression. There were no significant differences for the ICAM1 expression after 24 h. CX3CL1 is chemokines that can induce NK cell migration (El-Shazly et al., 2013; Ponzetta et al., 2013). It was interesting that FUS could enhance the CX3CL1 expression when combined with NK-92MI cells. The combined method could improve the CX3CL1 expression and further attract NK cells into tumor. However, the molecular mechanism of FUS and microbubbles were explored only initially in this study. Different time points after FUS treatment and transcriptome analysis need to be further explored to reveal the related mechanism in a much more comprehensive manner (Kovacs et al., 2017; Yang et al., 2017). It is important to figure out the key molecular basis for NK cells’ homing being assisted by FUS and to further improve the UTMD’s efficiency.

Other studies reported that SIR following increased BBB relied on microbubble doses, only pFUS+Definity at 100 μl/kg resulted in a clear activation of the NFκB signaling pathways, which is associated with inflammatory pathways, while 10 μl/kg did not elevate the NFκB pathways (El-Shazly et al., 2013). Thus, the microbubble doses may be another important factor that should be considered in improving the efficiency of UTMD (McMahon and Hynynen, 2017). Additionally, chemokine’s receptors modifying NK cells is also a novel strategy to improve the NK cells’ homing to tumors (Somanchi et al., 2012; Kremer et al., 2017).

It was consistent with our hypothesis that the FUS+NK-92MI group had a much better effect compared to the PBS group, which indicated that IL-2 played an important role in the function of NK cells *in vivo*. However, the FUS+NK-92MI group showed no significant differences compared to the NK-92MI group alone. When compared with Alkins’ research, the treatment therapy only succeeded when the tumor volume was small and treated in an intensive way early on (daily treatment in first 5 days) (Alkins et al., 2013b). Failures were observed when treatment was twice per week. These results indicated that adopting intensive early treatment may also be necessary for NK-92MI cells, even for NK cells with IL-2 genetic modification. When treating a small tumor burden, the ultrasound irradiation can irradiate most part of the tumor. However, when the tumor grew bigger, FUS could not irradiate the whole tumor, and necrosis without blood vessels in bigger tumors will also influence the NK cells’ delivery efficiency. Some studies developed a method to irradiate the whole tumor by irradiating every 1-mm grid extending over the entire tumor volume and improving the irradiation time to 5 min (Bandyopadhyay et al., 2016). The *in vitro* cellular cytotoxicity assay in this study showed that when the ratio of E/T was 2:1, the cytotoxicity of NK-92MI cells will work. Thus, further study should focus on the optimization of microbubble dosage, ultrasound irradiation time or treatment intensity to have therapeutic benefit.

## Conclusion

In conclusion, we demonstrated that the combination of FUS and microbubbles could increase the NK-92MI cells’ infiltration into tumors in this study. The combination of FUS and NK-92MI had a much better therapeutic effect when compared with the PBS group, but no superior therapeutic effect when compared with the NK-92MI group. In total, FUS and microbubbles can improve NK cells’ infiltration into tumors, but future work is still needed to improve NK-92MI cells’ delivery efficiency for solid tumor treatment.

## Ethics Statement

This study was carried out in accordance with the recommendations of Institutional Animal Care and Use Committee (IACUC) of Guangzhou Medical University.

## Author Contributions

CY, FY, and ZC designed the study. CY and MD performed the experimental work. CY were dealt the manuscript and data analysis, and drafted the manuscript. FY and ZC revised the manuscript.

## Conflict of Interest Statement

The authors declare that the research was conducted in the absence of any commercial or financial relationships that could be construed as a potential conflict of interest.
