# Corrigendum: Focused Ultrasound Improves NK-92MI Cells Infiltration Into Tumors

**DOI:** 10.3389/fphar.2019.00875

**Published:** 2019-08-06

**Authors:** Chaopin Yang, Meng Du, Fei Yan, Zhiyi Chen

**Affiliations:** ^1^Department of Ultrasound Medicine, Laboratory of Ultrasound Molecular Imaging, The Third Affiliated Hospital of Guangzhou Medical University, Guangzhou, China; ^2^Experimental Center, The Liwan Hospital of the Third Affiliated Hospital of Guangzhou Medical University, Guangzhou, China; ^3^Paul C. Lauterbur Research Center for Biomedical Imaging, Institute of Biomedical and Health Engineering, Shenzhen Institutes of Advanced Technology, Chinese Academy of Sciences, Shenzhen, China

**Keywords:** natural killer cells, IL-2, focused ultrasound, microbubbles, ovarian cancer

In the original article, the name of one author was missed in the reference for “Ponzetta, A., Sciume, G., Benigni, G., Antonangeli, F., Morrone, S., and Santoni, A. (2013). CX3CR1 regulates the maintenance of KLRG1+ NK cells into the bone marrow by promoting their entry into circulation. *J. Immunol.* 191, 5684–5694. doi: 10.4049/jimmunol.1300090.” It should be “Ponzetta, A., Sciume, G., Benigni, G., Antonangeli, F., Morrone, S., Santoni, A., et al. (2013). CX3CR1 regulates the maintenance of KLRG1+ NK cells into the bone marrow by promoting their entry into circulation. *J. Immunol*. 191, 5684–5694. doi: 10.4049/jimmunol.1300090.”

The authors apologize for this error and state that this does not change the scientific conclusions of the article in any way. The original article has been updated.

